# Evidence for Transmission of *Taenia solium* Taeniasis/Cysticercosis in a Rural Area of Northern Rwanda

**DOI:** 10.3389/fvets.2021.645076

**Published:** 2021-04-20

**Authors:** Lucrecia Acosta Soto, Lucy Anne Parker, María José Irisarri-Gutiérrez, Javier Arturo Bustos, Yesenia Castillo, Erika Perez, Carla Muñoz-Antoli, José Guillermo Esteban, Héctor Hugo García, Fernando Jorge Bornay-Llinares

**Affiliations:** ^1^Área de Parasitología del Departamento de Agroquímica y Medioambiente, Universidad Miguel Hernández de Elche, Alicante, Spain; ^2^Departamento de Salud Pública Historia de la Ciencia y Ginecología, Universidad Miguel Hernández de Elche, Alicante, Spain; ^3^Centro de Investigación Biomédica en Red de Epidemiología y Salud Pública (CIBERESP), Madrid, Spain; ^4^Área de Parasitología, Dpto. Farmacia y Tecnología Farmacéutica y Parasitología, Facultat de Farmàcia, Universitat de València, Valencia, Spain; ^5^Dpto. de Ciencias de la Salud, Facultad de Ciencias Biomédicas, Universidad Europea de Madrid, Madrid, Spain; ^6^Cysticercosis Unit, Instituto Nacional de Ciencias Neurológicas, Lima, Peru; ^7^Center for Global Health, Universidad Peruana Cayetano Heredia, Lima, Peru

**Keywords:** *Taenia solium*, taeniasis, cystcercosis, children, Gakenke, Rwanda

## Abstract

Cysticercosis is a parasitic infection caused by the metacestode larval stage (cysticercus) of *Taenia solium*. In humans, cysticercosis may infect the central nervous system and cause neurocysticercosis, which is responsible for over 50,000 deaths per year worldwide and is the major cause of preventable epilepsy cases, especially in low-income countries. Cysticercosis infection is endemic in many less developed countries where poor hygiene conditions and free-range pig management favor their transmission. A cross-sectional study was conducted in 680 children from a rural primary school in Gakenke district (Northern province of Rwanda). Stool samples were collected from participants and analyzed using the Kato-Katz method (KK), formol-ether concentration (FEC), and/or copro-antigen enzyme-linked immunosorbent assay (CoAg-ELISA) to detect taeniasis. Blood samples were collected and analyzed using enzyme-linked immunoelectrotransfer blot (EITB) and antigen enzyme-linked immunosorbent assay (Ag-ELISA) to detect human cysticercosis. The overall proportion of taeniasis positivity was 0.3% (2/680), and both cases were also confirmed by CoAg-ELISA. A total of 13.3% (76/572) of the children studied were positive to cysticercosis (*T. solium*-specific serum antibodies detected by EITB), of whom 38.0% (27/71) had viable cysticercus (*T. solium* antigens by Ag-ELISA). This study provides evidence of the highest cysticercosis prevalence reported in Rwanda in children to date. Systematic investigations into porcine and human cysticercosis as well as health education and hygiene measures for *T. solium* control are needed in Gakenke district.

## Introduction

In *Taenia solium* parasitic infections, humans are the only natural definitive host (taeniasis) while pigs are the intermediate hosts of larval stage (swine cysticercosis) (1). Human cysticercosis results when people become intermediate hosts after ingestion of microscopic viable eggs *via* the fecal–oral route from *T. solium* tapeworm carriers (2). The embryo is released (oncosphere), and it traverses the intestinal mucosa after ingestion. Later, it is transported by the circulatory system and dispersed by the organism producing cysts (cysticerci). The most common locations of cysts are the striated muscle, eyes, or heart tissue and central nervous system (3, 4). The clinical manifestations of cysticercosis are dependent on the number and location of cysticerci within the body (4). Some individuals with cysticercosis will exhibit or develop no symptoms (asymptomatic) or very mild symptoms. Many individuals with cysticercosis have central nervous system involvement (neurocysticercosis) resulting in headache, epileptic seizures, blindness, mental disturbance, and even death (3, 5). Neurocisticercosis (NCC) is the most common parasitic disease in the human nervous system and the most common cause of epilepsy in low-income countries (6).

Currently, cysticercosis is one of the 17 major Neglected Tropical Diseases (NTDs) identified by the WHO as a focus for research and control (7). It is widely prevalent where humans and domestic pig raising coexist. In many developing countries in Central and South America, Africa, and Asia, cysticercosis has major public health implications in humans and pigs (5, 8–11). It is in these places where poverty, poor education, lack of access to diagnosis, and limited management capacity, together with the absence of appropriate prevention measures and control strategies, make it highly endemic (11–13).

The distribution of *T. solium* taeniosis/cysticercosis in Africa is unclear but porcine and human cysticercosis are considered (hyper)-endemic in Central Africa (Rwanda, Burundi, the Democratic Republic of Congo, and Cameroon) (14, 15). In the last 20 years, pig production has increased significantly in the Eastern and Southern Africa (ESA) region, especially in rural, resource-poor, smallholder communities (11, 14). Several studies show a high prevalence of porcine cysticercosis in countries bordering Rwanda. In Uganda, prevalences ranging from 7.1 to 45% have been observed in urban areas in contrast to low percentages of 0.12–10.8% found in rural areas with an observed increment in recent years (9, 16–18). A prevalence of 41.2% has recently been reported in the Democratic Republic of Congo, where the overall prevalence of pigs with active cysticercosis did not significantly differ between the market and the village study sites but was much higher than previously observed by Chartier et al., in 1990 (19, 20). There are no current data for Burundi, but prevalence ranges from 2 to 39% were observed 20 years ago (21). In Tanzania, farm prevalence of porcine cysticercosis was between 17.4 and 18.2% in lingual examination or slaughter-slab prevalence by routine meat inspection respectively, while a maximum of 33.3% has been reported by cysticercal antigens by ELISA (Ag-ELISA) (22–29).

In contrast, Taeniasis has been poorly studied in humans from these countries (30). Eggs of *Taenia* sp. in feces have been reported in Uganda (31). Taeniasis prevalence ranges between 0 and 1.0% have been observed in schoolchildren of Burundi (21). However, prevalence of taeniasis ranging from 0.4 to 5.2% by the Kato-Katz technique or 2.3–5.2% by copro-Ag-ELISA has been estimated in Tanzania (32, 33).

Regarding human cysticercosis, one study estimated 21.6% prevalence of circulating antigen in the Democratic Republic of Congo (34). Several studies in human cysticercosis reveal the strong association between neurocysticercosis and epilepsy in these countries (35, 36). In Burundi, cysticercosis has been observed in 4.9–31.5% of epileptic patients, compared to 4.2% in controls (21, 37–39). On the other hand, a seroprevalence of up to 11.7% has been observed in epileptic patients and in 2.8% of controls (21). Cysticercosis was the cause of seizures in 25% of epileptic patients (38, 40). In Tanzania, cysticercosis prevalence of 16–17% was estimated and it was demonstrated that NCC contributed significantly to epilepsy in adults (32, 41–43). Given these data, it is likely that the situation in Rwanda is similar.

Rwanda has long since been known as a hyperendemic country for Taeniasis/cysticercosis. However, there are few research studies carried out in this country. Already in 1959, 20% of pigs were found to be infected with cysticercosis (44). From 2000 to 2011, pork production in Rwanda increased by 7.8% (45); however, an overall swine cysticercosis prevalence of 3.9% was found in farms, 9.2% in markets, and 4% in butchers (46). In 1956, the first case of human cysticercosis was reported in Rwanda (47). Since then, initial reports have reported isolated cases of disseminated ocular (48) and cutaneous cysticercosis (49, 50). In 1964, the presence of eggs of *Taenia* spp. in populations of the Batwa and Hutu tribes in the Northern and Southern regions of the country was detected (51). Later, *T. solium* cysticerci was detected in 7% of 300 autopsies in Butare (52). In Kigali and Butare, 21 and 21.8% of people with epilepsy are estimated to be seropositive for cysticercosis, respectively (53, 54). To date, there have been no studies in children.

During a coproparasitological study in the school population of Northern Rwanda, eggs from tapeworms were detected in two schoolchildren. After collecting the strobila, the morphology of mature proglottids allowed us to identify the species as *T. solium* in both cases. This observation motivated us to investigate the presence of cysticercosis in the school population of Nemba, with the prospect of introducing control activities.

## Methods

### Study Area

**Figure 1:**
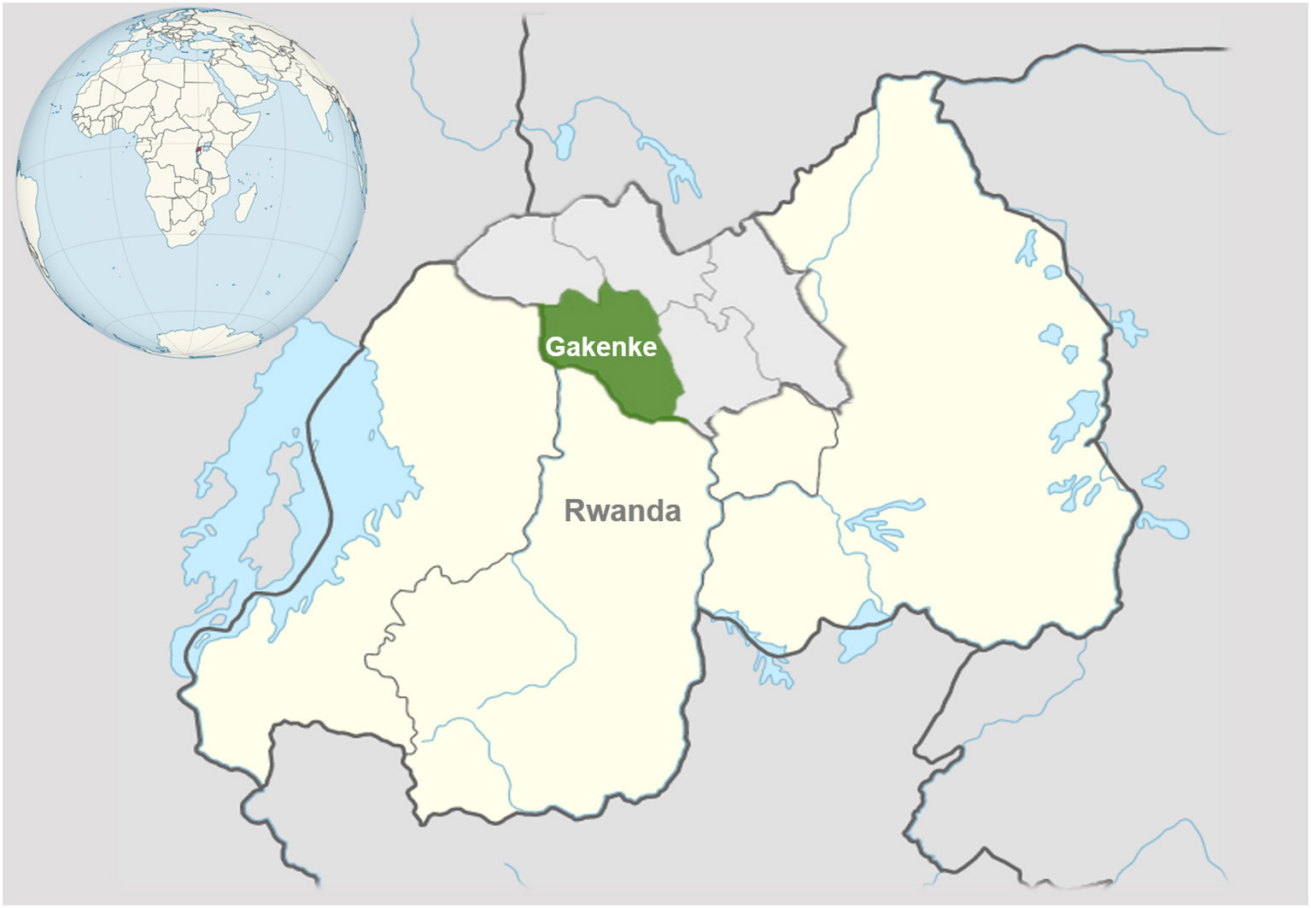
Study location map. District of Gakenke, Northern Province, Rwanda.

Rwanda is a small landlocked country in Central Africa, bordering Uganda to the North, Burundi to the South, Democratic Republic of the Congo to the West, and Tanzania to the East. The estimated population in 2012 was 10.4 million people, thus supporting the densest population in continental Africa, with most of the population engaged in subsistence agriculture (55). A verdant country of fertile and hilly terrain with altitudes varying from 950 m to 4,519 m, the small republic bears the title “Land of a Thousand Hills.” The District of Gakenke is one of the five districts of the Northern Province of Rwanda. This district is divided into 19 administrative sectors made of 97 cells, 617 villages, and 345,487 inhabitants living in a total area of 104 km^2^ with a population density of 473/km^2^ (56). Figure 1 shows a schematic map of the study area.

Gakenke district is characterized, in general, by high inclined hills separated by rivers and marshlands. The climate in Gakenke district is generally the type of humid climate with the average annual temperature varying between 16.0 and 29.0°C. The rainfalls are relatively abundant with a scale between 1,100 and 1,500 mm per year (56).

### Population

**Figure 2:**
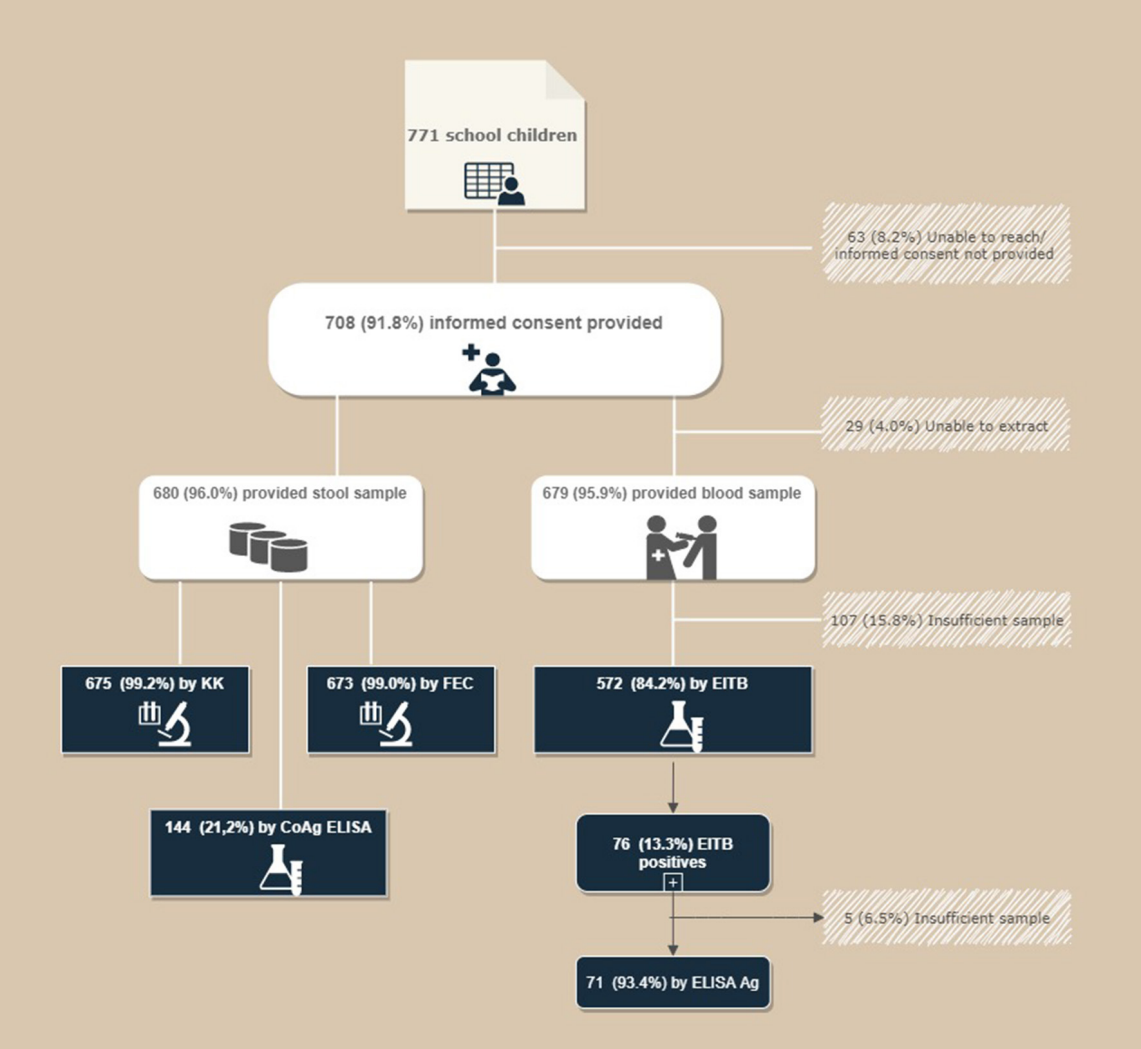
Flow chart for the recruitment of patients.

Between July and September 2011, the Parasitology Area of University Miguel Hernández de Elche (UMH; Spain) conducted a cross-sectional study in Nemba school I, Gakenke district of Rwanda, as part of an initial phase of a school health program. A total of 771 schoolchildren (371 girls and 400 boys) attended primary education (2nd to 6th grade) and were eligible for inclusion in the study. A total of 708 students (357 boys and 351 girls aged between 6 and 18 years of age; mean ± SD = 11.00 ± 2.33 years) were eligible for the study after their parents or guardians provided informed consent. A total of 680 answered the questionnaire and provided stool sample, while 572 provided blood sample (see Figure 2).

### Sampling and Parasitological Tests

After collection, fresh feces samples were processed and quantified *in situ* (UMH headquarters in Nemba, Rwanda) by the method described by Kato-Katz following WHO recommendations (57). A 41.7-mg sample of fresh feces was used as template according to the Helm teste® manufacturer's recommendations (Bio-Manguinhos/Fiocruz, Brazil). If taeniid eggs were observed, 2 g of Niclosamide and 1 ml of Duphalac® oral solution (667 mg/ml of lactulose) was administered as a laxative to recover the adult where possible.

Additionally, part of each stool sample was preserved in 10% buffered formalin (1:3) (40-ml Falcon tube) and another part was preserved in ethanol 70% (2-ml tube). Sera samples were obtained from whole blood collected of every child by centrifugation and stored at 4°C in Nemba's Hospital until sent to UMH, Spain. Later, all samples were sent to UMH and stored at −20°C until processing. Formalin-embedded samples were sent to the Department of Parasitology of University of Valencia (Spain) where a formol-ether concentration was realized (58). Finally, tree drops of sediment with Lugol's iodine solution were analyzed under a microscope. Sera and fecal samples preserved in ethanol 70% were subsequently sent to the diagnostic laboratory of the Center for Global Health of the Universidad Peruana Cayetano Heredia (UPCH, Lima, Peru) for processing by *T. solium*-specific tests [Coproantigen ELISA, serum antigen detection (Ag-ELISA), and serum antibody detection by enzyme-linked immunoelectrotransfer blot (EITB) assay using lentil lectin-purified parasitic glycoprotein antigens].

### Taeniasis: Coproantigen Detection by ELISA (CoAg-ELISA)

In an attempt to find additional carriers of adult *T. solium* worms, a subgroup of 144 fecal samples (~21% of the total) were also screened by coproantigen ELISA (59, 60). Aliquots (1.5 ml) of the stool supernatant were used for CoAg-ELISA after centrifugation at room temperature at 3,200 g for 10 min. The CoAg-ELISA technique was performed as described by Allan et al., by using hyperimmune rabbit anti-*T. solium* IgG as the capture antibody and peroxidase-labeled goat anti-*T. solium* IgG as a conjugate. Processed samples were read with a spectrophotometer (Molecular Devices Inc., Sunnyvale, CA) at 650 nm. Using a known positive pool (P1), we calculated the percentage of positivity (PP) as [optical density (OD) of the sample]/(OD of P1) 100, in order to increase the comparability of the results between plates. A cutoff was determined using a receiver operating characteristic (ROC) curve (61).

### Cysticercosis-Specific Tests

#### Anti-*T. solium* Antibody Detection

A total of 572 sera samples were processed by EITB. The methodology used to perform the test was the same described by Tsang et al. (62) and González et al. (63). An immunoblot of seven cysticercus glycoproteins (GP50, GP42-39, GP24, GP21, GP18, GP14, and GP13), purified by lentil lectin-purified chromatography, gives close to 100% specificity and a sensitivity varying from 70 to 90% (62). This EITB has been widely used for the diagnosis of cysticercosis in human and pig serum samples (64).

#### *T. solium* Antigen Detection by ELISA

In order to demonstrate the presence of active infections, an anti-*T. solium* antibody-positive subgroup of children (71 EITB positive samples) were processed in quest by ELISA, to detect circulating cysticercal antigen in the serum (65, 66). A mAb-based ELISA for the detection of circulating antigens was used to detect circulating parasite antigen as described by Brandt et al. in 1992 (67) and later adapted by Van Kerckhoven et al. and Dorny et al. (68, 69). The assay uses Nunc MaxiSorp plates sensitized with a trapping mAb (B158C11A10) in bicarbonate buffer at 5 μg/ml. After blocking, serum samples (pretreated with 5% trichloroacetic acid to break existing immune complexes) are added, followed by the second mAb (B60H8A4-BIOT), streptavidin, o-phenylenediamine (OPD/H2O2) as substrate/chromogen, and incubated in the dark for 15 min. The reaction is stopped with H_2_SO_4_ and plates are read at 490/650 nm. To minimize inter-plate variation, we used antigen ratio instead of a raw value of optic density (OD). The antigen ratio is estimated by dividing the OD of the tested sample with the mean of eight negative samples plus three standard deviations (70).

### Questionnaire and Anthropometric Measurements

A questionnaire was concurrently conducted while blood and stool samples were taken. The children were interviewed, and basic socio-demographic data (age, sex, residence) were recorded as well as specific questions about their living conditions and behaviors. Each child was assigned a unique ID code and removed their shoes so that we could measure their height (in cm) and weight (in kg).

### Statistical Analyses

The data were identified and entered into a Microsoft Office Excel 2010 spreadsheet (Microsoft). Statistical analysis was performed using Stata SE 15.0 (StataCorp LLC, USA). We calculated the frequencies of categorical variables and made comparisons using Pearson's Chi-squared-test. Missing values were excluded for the comparisons. Continuous variables were described with means and standard deviations.

## Results

### Taeniasis Intestinal and Species Identification

The coproparasitological analysis revealed an overall parasitism of 94.9% (639/673) by formol-ether concentration and a helminth parasitism percentage of 31.2% (211/675) by the Kato-Katz technique. In two children [2/680 (0.3%)], eggs of taeniid tapeworm were identified by both methods: Kato-Katz technique [2/675 (0.3%)] and formol-ether concentration [2/673 (0.31%)].

After parental consent, 2 g of Niclosamide and 1 ml of Duphalac® oral solution (667 mg/ml of lactulose) as a laxative were administered and the feces were collected. Two strobiles were recovered from both children and the morphologic study under a magnifying glass after injection with India ink revealed <12 uterine branches compatible with *T. solium*-proglottid demonstrating current tapeworm infection in both children.

Moreover, 144 samples were tested by coproantigen ELISA detection and 2/144 (1.4%) samples had positive results. The positive samples obtained matched the positive samples obtained by Kato-Katz and formol-ether concentration techniques.

### Cysticercosis

EITB analysis of a total of 572 sera samples revealed that 13.3% of the students (76/572) presented reactivity to one or more of the specific glycoproteins of *T. solium*, of whom 46.0% (35/76) showed reactivity to three or more bands. A total of 7 of the 76 positive samples showed a single GP 50 band reaction, whereas in 9 of the positive samples, no GP 50 band was observed.

Exposure to *T. solium* was more frequent among girls than boys (*p* = 0.014, Table 1) and was especially prevalent in the Mucaca area of Nemba where 21.3% of the children had anti-*T. solium* antibodies (*p* < 0.001). Children of all ages and in all classes had positive EITB results, and there was no obvious relationship with domestic animal rearing. The majority of the children came from homes that used a water pump as their main water source and had a latrine. Consumption of meat was significantly associated with *T. solium* exposure. The highest levels of exposure were among children that reported consuming meat once a month (15.6%) or once a year (19.2%); 13.1% of children who reported that they always washed their hands after going to the toilet had anti-*T. solium* antibodies, and this was not statistically different from children that reported they sometimes or never washed their hands (12.8 and 14.3%, respectively).

**Table 1:** Results of the cysticercosis-specific tests conducted according to characteristics of the children.

**Characteristics**	**EITB**				**Ag-ELISA**	
	**Positive**		**3 or more bands**			
	***n*/total (%)**	***p-*value^a^**	***n*/positive EITB (%)**	***p*-value^a^**	***n*/positive EITB (%)**	***p-*value^a^**
**Sex**		0.019		0.598		0.948
Female	48/289 (16.6)		21/48 (43.7)		18/47 (38.3)	
Male	28/282 (9.9)		14/28 (50.0)		9/24 (37.5)	
No data	0/1 (0.0)		0/1 (0.0)		0/0 (0.0)	
**Age**		0.509		0.606		0.674
6–9 years	20/155 (13.0)		11/20 (55.0)		8/19 (42.1)	
10–14 years	46/363 (12.7)		20/46 (43.5)		15/44 (34.1)	
15–18 years	8/42 (19.0)		3/8 (37.5)		3/6 (50.0)	
No data	2/12 (16.7)		1/2 (50.0)		0/2	
**Sector**		<0.001		0.711		0.401
**Nemba**						
Buranga	3/46 (6.5)		2/3 (66.7)		0/3 (0.0)	
Gahinga	7/70 (10.0)		3/7 (42.8)		3/6 (50.0)	
Gisozi	13/202 (6.4)		5/13 (38.5)		3/12 (25.0)	
Mucaca	46/216 (21.3)		24/46 (52.2)		19/43 (45.2)	
**Gakenke**						
Rusagara	4/25 (16.0)		1/4 (25.0)		11/4 (27.5)	
No data	3/13 (23.1)		0/3 (0.0)		1/3 (33.3)	
**School class**		0.204		0.545		0.650
P2	15/150 (10.0)		7/15 (46.7)		7/14 (50.0)	
P3	17/114 (14.9)		10/17 (58.8)		6/16 (37.5)	
P4	24/131 (18.2)		8/24 (33.3)		7/23 (30.4)	
P5	15/116 (12.9)		7/15 (46.7)		6/13 (46.1)	
P6	5/61 (8.2)		3/5 (60.0)		1/5 (20.0)	
**Domestic animals**		0.270		0.299		0.415
Pigs	1/28 (3.6)		0/1 (0.0)		1/1 (100.0)	
Other animals^b^	64/459 (13.9)		31/64 (48.4)		21/59 (35.6)	
None	8/68 (11.8)		2/8 (25.0)		3/8 (37.5)	
Don't know/no answer provided	1/5 (20.0)		1/1 (100.0)		1/1 (100.0)	
No data	2/12 (16.7)		1/2 (50.0)		1/2 (50.0)	
**Water source**		0.515		0.809		0.077
Piped water	5/58 (8.6)		2/5 (40.0)		0/5 (0.0)	
Water pump/fountain	68/496 (13.7)		31/68 (45.6)		25/63 (39.7)	
Rivers/puddles	0/1 (0.0)		0/0 (0.0)		0/0 (0.0)	
Don't know/no answer provided	1/5 (20.0)		1/1 (100.0)		1/1 (100.0)	
No data	2/12 (16.7)		1/2 (50.0)		1/2 (50.0)	
**Toilet**		0.432		NA		NA
WC	0/10 (0.0)		0/0 (0.0)		0/0 (0.0)	
Private latrine	72/542 (13.3)		32/72 (44.4)		25/67 (37.3)	
Shared latrine	0/1 (0.0)		0/1 (0.0)		0/0 (0.0)	
Don't know/no answer provided	2/7 (28.6)		2/2 (100.0)		1/2 (50.0)	
No data	2/12 (16.7)		1/2 (50.0)		1/2 (50.0)	
**How often do you eat meat?**		0.003		0.070		0.818
Once a week	10/159 (6.3)		7/10 (70.0)		2/9 (22.2)	
Once a month	32/205 (15.6)		12/32 (37.5)		11 /30 (36.7)	
Once a year	27/141 (19.2)		11/27 (40.7)		10/25 (40.0)	
Never	3/44 (6.8)		3/3 (100.0)		1/3 (33.3)	
Don't know/no answer provided	2/11 (18.2)		1/2 (50.0)		2/2 (100.0)	
No data	2/12 (16.7)		1/2 (50.0)		1/2 (50.0)	
**How often do you wash your hands after going to the toilet?**		0.935		0.367		0.788
Always	26/198 (13.1)		9/26 (34.6)		10/24 (41.7)	
Sometimes	34/266 (12.8)		18/34 (52.9)		12/34 (35.3)	
Never	13/91 (14.3)		6/13 (46.1)		3/10 (30.0)	
Don't know/no answer provided	1/5 (20.0)		1/1 (100.0)		1/1 (100.0)	
No data	2/12 (16.7)		1/2 (50.0)		1/2 (50.0)	
**Total**	**76/572 (13.3)**		**35/76 (46.0)**		**27/71 (38.0)**	

*EITB, Anti-T. solium antibodies detection by enzyme-linked immunoelectrotransfer blot; Ag-ELISA, T. solium antigen detection by ELISA*.

a *p-value from a Chi squared-test comparing answer categories, excluding missing or unknown values*.

b *Other animals: Cows, sheep, goats, rabbits, and chicken*.

*Taenia solium* antigens were found in 38% (27/71) of children with anti-*T. solium* antibodies suggesting active infection. In addition, 14 of the 27 positive samples for antigen presented more than three bands by EIBT-test. None of the variables considered were associated with antigen presence among seropositive children (Table 1).

## Discussion

The present study demonstrates a substantial seroprevalence of cysticercosis in children of the Northern Province of Rwanda. The results suggest that more than of 13% of this group of children had been exposed to *T solium*. In addition, among the reactive individuals, 46% had three or more reactive bands by EITB, which is highly suggestive of established cysticercosis infection. Furthermore, of the children with antibodies to cysticercosis, 38% were reactive to the detection of circulating antigen by ELISA, indicating the presence of active cases of neurocysticercosis, as previously demonstrated (65).

In 1982, Fain et al. said that *T. solium* was the most prevalent tapeworm in Rwanda (47). In previous studies in Rwanda, soil-transmitted helminths and schistosomiasis but no tapeworms were found. Since 1964, human taeniosis has not been detected in the country (51). However, analysis based on both microscopy or coproantigen detection of human fecal material cannot identify the species level, and the possibility of *T. saginata* presence cannot be ruled out. Other studies showed high parasitic burden strongly associated with drinking any kind of water (71–73).

The finding of eggs of *Taenia* spp. in two schoolchildren motivated us to deepen the search for associated human cysticercosis. Both children were positive based on microscopy (KK and FEC) and coproantigen detection (copro-Ag-ELISA).

In spite of false positives reported by coproantigen test (74), a high positivity value is highly predictive of a true positive (61). No cross-reactions were observed with other intestinal parasites (protozoa and helminths) present in the children. In this study, the microscopy and coproantigen detection assays had the same sensitivity as has been reported by other authors (59, 75). Our work showed some limitations because we were unable to carry out the coproantigen study on all samples, and the sample of children included came from a single school in the Gakenke district. However, participation was high, and with this study, we can confirm the presence of tapeworm carriers in this area. Additionally, we identified morphologically both adult specimens. Identification to species level in this country or bordering countries except in Tanzania has never been done (33).

To our knowledge, this is the first report of cysticercosis in schoolchildren in Rwanda. In cysticercosis surveys and immunodiagnostic tools applied on humans, serum samples are useful in estimating the prevalence and identifying of *T. solium* infection in the field. *T. solium* antigen in serum could predict the presence of viable brain parasites in patients with apparently calcified cysticercosis only (70). Antibody response in *T. solium* infection in field conditions was found to be a major contributor to the overestimation of the prevalence of cysticercosis in endemic areas (4, 76). The frequency found in these children was higher than prior literature reports in Butare, where 7% of autopsies were positive for cysticercosis (52). Furthermore, 21 and 21.8% of patients with epilepsy in Kigali and southern Rwanda, respectively, are positive for cysticercosis (50, 53, 54). None of the previous studies were performed in children. In our study, it is important to highlight the early age of the population studied, since it is known that the prevalence of cysticercosis in a region increases with the age range studied. We can deduce that the seroprevalence in the adult population is likely to be higher than observed here.

The highest proportion of positive schoolchildren was observed in the same sector where tapeworm carriers were located. We observed small-scale swine production system in Mucaca (personal observation).

The financial losses due to human cysticercosis are very difficult to estimate but are certainly exceeded by the social impact of the disease, especially because of the particular perception of epilepsy in many African communities (14). Conditions like neurocysticercosis are better prevented than treated, because it has the potential to make a healthy person invalid. If the treatment is delayed, there can be irreversible brain damage.

In our study, no association was observed in frequency of hand washing after going to the toilet. However, in our study, close contact with pigs and use of latrines as possible risk factors for cysticercosis were not significantly associated with positive cases as observed in Tanzania (43). The absence of association may be explained by the level of widespread poverty in this area. Interestingly, meat consumption showed a significant association with cysticercosis exposure, but it was not a dose response. It is possible that people with limited resources cannot afford quality meat in these rural areas. Children who reported consuming meat once a month or once a year had higher exposure rates than those who reported consuming meat weekly, but it is possible that here we are seeing the impact of socio-economic status of the children rather than meat consumption itself. It has been shown that vegetarians and other people who do not eat pork can acquire cysticercosis through fecal–oral contamination with *T. solium* eggs from tapeworm carriers (77). Unhygienic sanitary conditions such as limited use or absence of latrines are prevalent in rural areas of Africa where pigs are raised (14, 78, 79). Informally marketed foods, clandestine slaughtering of pigs, lack of trained and qualified meat inspectors, lack of detection and treatment of *T. solium* carriers, and consumption of undercooked or insufficiently cooked pork could be risk factors for taeniosis/cysticercosis among consumers (22, 26, 35).

A nutritional survey and coproparasitological examination was being carried out at the Nemba 1 School, Gakenke district, Rwanda, when two samples harboring *Taenia* spp. eggs were found (unpublished results). It was impossible to obtain more samples or additional information that would allow the analysis of other risk factors or possible variables related to clinical manifestations of the infection. For the reasons stated, we decided to carry out the present study with the sole objective of investigating the presence of infection and transmission of *T. solium* in the community. The results obtained confirm the presence and transmission of *T. solium* and support the need to design more extensive future studies.

## Conclusions

Overall, our study shows the presence and transmission of *T. solium* in Gakenke district. The high seroprevalence found in this area allows it to be considered hyperendemic for cysticercosis. To avoid neurological consequences, systematic investigations into porcine and human cysticercosis as well as health education and hygiene measures for *T. solium* control are needed. It is necessary to evaluate the clinical status of schoolchildren involved, carry out a study of contacts, and implement actions for the control and prevention of the disease.

## Data Availability Statement

All data analyzed during this study are included in this published article. The raw data or any further information supporting the conclusions of this article will be made available by the corresponding author on reasonable request.

## Ethics Statement

The studies involving human participants were reviewed and approved by Experimental Research Commission on Ethics from University Miguel Hernández de Elche (Spain) (Ref: DF-MPA-001-11). Written informed consent to participate in this study was provided by the participants' legal guardian/next of kin.

## Author Contributions

FB-L, HG, and JGE contributed to conception and design of the study. LA, LP, and FB-L supervised and coordinated the field study. LA, MI-G, and FB-L carried out the coproparasitological analysis in the field. CM-A and JGE coordinated the coproparasitological procedures. JB, YC, and EP coordinated and performed the serological and coproantigen tests. LA and LP organized the database-analyzed data, realized formal analysis, and interpretation of data. LA, LP, and HG wrote the first draft of the manuscript. FB-L critically reviewed it and contributed to draft design. All authors contributed to manuscript revision, read, and approved the submitted version.

## Conflict of Interest

The authors declare that the research was conducted in the absence of any commercial or financial relationships that could be construed as a potential conflict of interest.

## References

[1] Flisser A. Taeniasis and cysticercosis due to *Taenia solium*. Prog Clin Parasitol. (1994) 4:77–116. 7948938

[2] García HH Del Brutto OH. Imaging findings in neurocysticercosis. Acta Tropica. (2003) 87:71–8. 10.1016/S0001-706X(03)00057-3 12781380

[3] White AC. Neurocysticercosis: updates on epidemiology, pathogenesis, diagnosis, and management. Annu Rev Med. (2000) 51:187–206. 10.1146/annurev.med.51.1.187 10774460

[4] Garcia HH Gonzalez AE Gilman RH. Taenia solium Cysticercosis and its Impact in Neurological Disease. Clin Microbiol Rev. (2020) 33:e00085-19. 10.1128/CMR.00085-19 32461308 PMC7254859

[5] Garcia HH Del Brutto OH. *Taenia solium* cysticercosis. Infect Dis Clin North Am. (2000) 14:97–119. 10.1016/S0891-5520(05)70220-8 10738675

[6] Preux PM Druet-Cabanac M. Epidemiology and aetiology of epilepsy in sub-Saharan Africa. Lancet Neurol. (2005) 4:21–31. 10.1016/S1474-4422(04)00963-9 15620854

[7] WHO. First WHO Report on Neglected Tropical Diseases: Working to Overcome the Global Impact of Neglected Tropical Diseases. Geneva: World Health Organisation (2010). p. 1–184.

[8] Sarti E Rajshekhar V. Measures for the prevention and control of *Taenia solium* taeniosis and cysticercosis. Acta Tropica. (2003) 87:137–43. 10.1016/S0001-706X(03)00034-2 12781388

[9] Nsadha Z Thomas LF Fèvre EM Nasinyama G Ojok L Waiswa C. Prevalence of porcine cysticercosis in the Lake Kyoga Basin, Uganda. BMC Vet Res. (2014) 10:239. 10.1186/s12917-014-0239-y 25286901 PMC4195862

[10] Wu HW Ito A Ai L Zhou XN Acosta LP Lee Willingham A. Cysticercosis/taeniasis endemicity in Southeast Asia: current status and control measures. Acta Trop. (2017) 165:121–32. 10.1016/j.actatropica.2016.01.013 26802488

[11] Phiri IK Ngowi H Afonso S Matenga E Boa M Mukaratirwa S. The emergence of *Taenia solium* cysticercosis in Eastern and Southern Africa as a serious agricultural problem and public health risk. Acta Tropica. (2003) 87:13–23. 10.1016/S0001-706X(03)00051-2 12781374

[12] Willingham AL Engels D. Control of *Taenia solium* cysticercosis/taeniosis. Adv Parasitol. (2006) 61:509–66. 10.1016/S0065-308X(05)61012-3 16735172

[13] O'Neal SE Moyano LM Ayvar V Gonzalvez G Diaz A Rodriguez S. Geographic correlation between tapeworm carriers and heavily infected cysticercotic pigs. PLoS Negl Trop Dis. (2012) 12:1953. 10.1371/journal.pntd.0001953 23285305 PMC3527375

[14] Zoli A Shey-Njila O Assana E Nguekam JP Dorny P Brandt J. Regional status, epidemiology and impact of *Taenia solium* cysticercosis in Western and Central Africa. Acta Tropica. (2003) 87:35–42. 10.1016/S0001-706X(03)00053-6 12781376

[15] Braae UC Saarnak CFL Mukaratirwa S Devleesschauwer B Magnussen P Johansen MV. *Taenia solium* taeniosis/cysticercosis and the co-distribution with schistosomiasis in Africa. Parasites Vectors. (2015) 8:323. 10.1186/s13071-015-0938-7 26065414 PMC4465723

[16] Waiswa C Fèvre EM Nsadha Z Sikasunge CS Willingham AL. Porcine cysticercosis in Southeast Uganda: seroprevalence in Kamuli and Kaliro Districts. J Parasitol Res. (2009) 2009:1–5. 10.1155/2009/375493 20721330 PMC2915645

[17] Kisakye J Mal S. Short communication. Cysticcrcus ccllulosae in pigs slaughtered i~ and around Kampala city. Uganda J Agric Sci. (2002) 7:23–4. Available online at: https://www.ajol.info/index.php/ujas/article/view/128878

[18] Kungu JM Dione MM Ejobi F Harrison LJS Poole EJ Pezo D. Sero-prevalence of *Taenia* spp. cysticercosis in rural and urban smallholder pig production settings in Uganda. Acta Trop. (2017) 165:110–5. 10.1016/j.actatropica.2016.01.016 26801882

[19] Chartier C Mutesi U Ndakala NO. Les helminthes du porc domestique en Ituri, Haut-Zaïre. Ann Soc Belg Med Trop. (1990) 70:213–25. 2241308

[20] Praet N Kanobana K Kabwe C Maketa V Lukanu P Lutumba P. *Taenia solium* cysticercosis in the democratic Republic of Congo: how does pork trade affect the transmission of the parasite?. PLoS Negl Trop Dis. (2010) 4:e817. 10.1371/journal.pntd.0000817 20838646 PMC2935392

[21] Newell E Vyungimana F Geerts S Van Kerckhoven I Tsang VCW Engels D. Prevalence of cysticercosis in epileptics and members of their families in Burundi. Trans R Soc Trop Med Hyg. (1997) 91:389–91. 10.1016/S0035-9203(97)90251-0 9373628

[22] Boa ME Mahundi EA Kassuku AA Willingham AL Kyvsgaard NC. Epidemiological survey of swine cysticercosis using ante-mortem and post-mortem examination tests in the southern highlands of Tanzania. Vet Parasitol. (2006) 139:249–55. 10.1016/j.vetpar.2006.02.012 16647211

[23] Mellau BL Nonga HE Karimuribo ED. Slaughter stock abattoir survey of carcasses and organ/offal condemnations in Arusha region, northern Tanzania. Trop Anim Health Prod. (2011) 43:857–64. 10.1007/s11250-010-9773-1 21188519

[24] Mkupasi EM Ngowi HA Nonga HE. Prevalence of extra-intestinal porcine helminth infections and assessment of sanitary conditions of pig slaughter slabs in Dar es Salaam city, Tanzania. Trop Anim Health Prod. (2011) 43:417–23. 10.1007/s11250-010-9708-x 20938736

[25] Mkupasi EM Ngowi HA Sikasunge CS Leifsson PS Johansen MV. Efficacy of ivermectin and oxfendazole against *Taenia solium* cysticercosis and other parasitoses in naturally infected pigs. Acta Trop. (2013) 128:48–53. 10.1016/j.actatropica.2013.06.010 23806569

[26] Komba EVG Kimbi EC Ngowi HA Kimera SI Mlangwa JE Lekule FP. Prevalence of porcine cysticercosis and associated risk factors in smallholder pig production systems in Mbeya region, southern highlands of Tanzania. Vet Parasitol. (2013) 198:284–91. 10.1016/j.vetpar.2013.09.020 24139481

[27] Ngowi HA Kassuku AA Maeda GEM Boa ME Carabin H Willingham AL. Risk factors for the prevalence of porcine cysticercosis in Mbulu District, Tanzania. Vet Parasitol. (2004) 120:275–83. 10.1016/j.vetpar.2004.01.015 15063938

[28] Yohana C Mwita C Nkwengulila G. The Prevalence of Porcine Cysticercosis and Risk Factors for Taeniasis in Iringa Rural District. (2013). Available online at: http://repository.udsm.ac.tz:8080/xmlui/handle/123456789/1505 (accessed December 14, 2020).

[29] Wilson RT Swai E. A review of pig pathology in Tanzania. Trop Anim Health Prod. (2013) 45:1269–75. 10.1007/s11250-013-0426-z 23733144

[30] Braae UC Harrison W Lekule F Magnussen P Johansen MV. Feedstuff and poor latrines may put pigs at risk of cysticercosis—A case-control study. Vet Parasitol. (2015) 214:187–91. 10.1016/j.vetpar.2015.08.009 26304509

[31] Kabatereine NB Tukahebwa EM Brooker S Alderman H Hall A. Epidemiology of intestinal helminth infestations among schoolchildren in southern Uganda. East Afr Med J. (2001) 78:283–6. 10.4314/eamj.v78i6.9019 12002102

[32] Mwanjali G Kihamia C Kakoko DVC Lekule F Ngowi H Johansen MV. Prevalence and risk factors associated with human *Taenia Solium* infections in Mbozi District, Mbeya Region, Tanzania. PLoS Negl Trop Dis. (2013) 7:2102. 10.1371/journal.pntd.0002102 23516650 PMC3597471

[33] Eom KS Chai J-Y Yong T-S Min D-Y Rim H-J Kihamia C. Morphologic and genetic identification of taenia tapeworms in Tanzania and DNA genotyping of *Taenia solium*. Korean J Parasitol. (2011) 49:399. 10.3347/kjp.2011.49.4.399 22355207 PMC3279678

[34] Kanobana K Praet N Kabwe C Dorny P Lukanu P Madinga J. High prevalence of *Taenia solium* cysticerosis in a village community of Bas-Congo, Democratic Republic of Congo. Int J Parasitol. (2011) 41:1015–8. 10.1016/j.ijpara.2011.06.004 21763695

[35] Makita K Fèvre EM Waiswa C Kaboyo W Eisler MC Welburn SC. Evidence-based identification of the most important livestock related zoonotic diseases in Kampala, Uganda. J Vet Med Sci. (2011) 73:49. 10.1292/jvms.11-0049 21467754

[36] Ngugi AK Bottomley C Kleinschmidt I Wagner RG Kakooza-Mwesige A Ae-Ngibise K. Prevalence of active convulsive epilepsy in sub-Saharan Africa and associated risk factors: cross-sectional and case-control studies. Lancet Neurol. (2013) 12:253–63. 10.1016/S1474-4422(13)70003-6 23375964 PMC3581814

[37] Nzisabira L Nsengiyumva G Bouteille B Ndayiragije A Niyongabo T Bigirimana V. Cysticercosis in the province of Kayanza (Burundi). Bull Soc Pathol Exot. (1992) 85:374–7. 1292797

[38] Nsengiyumva G Druet-Cabanac M Ramanankandrasana B Bouteille B Nsizabira L Preux P-MM. Cysticercosis as a major risk factor for epilepsy in Burundi, East Africa. Epilepsia. (2003) 44:950–5. 10.1046/j.1528-1157.2003.55302.x 12823579

[39] Prado-Jean A Kanobana K Druet-Cabanac M Nsengyiumva G Dorny P Preux PM. Combined use of an antigen and antibody detection enzyme-linked immunosorbent assay for cysticercosis as tools in an epidemiological study of epilepsy in Burundi. Trop Med Int Heal. (2007) 12:895–901. 10.1111/j.1365-3156.2007.01860.x 17596257

[40] Diagana M Nsengiyumva G Tuillas M Druet-Cabanac M Bouteille B Preux PM. Électroencéphalogrammes réalisés chez 250 patients épileptiques dans une zone d'endémie cysticerquienne au Burundi. Neurophysiol Clin. (2005) 35:1–10. 10.1016/j.neucli.2004.12.002 15808962

[41] Mwang'onde BJ Nkwengulila G Chacha M. The risk factors for human cysticercosis in Mbulu District, Tanzania. Onderstepoort J Vet Res. (2014) 81:5. 10.4102/ojvr.v81i2.719 25005750

[42] Winkler AS Willingham AL Sikasunge CS Schmutzhard E. Epilepsy and neurocysticercosis in sub-Saharan Africa. Wiener Klinische Wochenschrift. (2009) 121:3–12. 10.1007/s00508-009-1242-3 19915809

[43] Blocher J Schmutzhard E Wilkins PP Gupton PN Schaffert M Auer H. A cross-sectional study of people with epilepsy and Neurocysticercosis in Tanzania: clinical characteristics and diagnostic approaches. PLoS Negl Trop Dis. (2011) 5:1185. 10.1371/journal.pntd.0001185 21666796 PMC3110162

[44] Thienpont D De Keyser J Vandervelden M Kageruka P. La Cysticercose ce're'brale du porc. Me'd Trop. (1959) 39:507–14. 13837798

[45] Food and Agriculture Organization of the United Nations. Food Outlook: Biannual Report on Global Food Markets. Rome: ASHA Lead (2014). p. 18–9. Available online at: http://www.fao.org/3/i4136e/i4136e.pdf

[46] Mushonga B Habarugira G Birori A Kandiwa E Samkange A Bhebhe E. An epidemiological survey of the magnitude and local perceptions of porcine cysticercosis by two methods in Nyaruguru district, Rwanda. Vet Parasitol Reg Stud Rep. (2018) 14:18–24. 10.1016/j.vprsr.2018.07.010 31014726

[47] Fain A Musafili I. Verminoses. In: Meheus A Butera S Eylenboceh W Gatera G Kivits M Musafili I editors. Sante et Maladies au Rwanda. Bruxelles: Administration Generaale de la Coopération au Développement (1982). p. 314–29.

[48] Vanderick F Fain A Langi S Vanbalen H. Deux noveaux cas de c'enurose humaine 'a Taenia brauni au Rwanda, avec une localisation orbitaire du c'enure. Ann Soc Belg Med Trop. (1920) 44:1077–9. 14323527

[49] Gascón J Corachan M Ramiréz J. 1989 Gascón_Cysticercosis_5 cases Rwanda. Med Trop. (1989) 49:77–80. 2725249

[50] Tuan J Kailani L Ngabitsinze P Umuganwa S Munyaneza F Musoni E. Disseminated cysticercosis in rwanda-case report of a patient presenting with difficulty with walking and skin nodules. Rwanda Med J. (2020) 77:30–33. Available online at: https://www.rwandamedicaljournal.org/uploads/1/2/2/1/122149944/cr.19.19.pdf

[51] Scaglia M Gatti S Malfitano A Strosselli M Brusti R. Incidence of intestinal parasitosis among the Batwa and Hutu pygmy tribes of Rwanda. Bull Soc Pathol Exot Fil. (1983) 76:818–24. 6671271

[52] Vanderick FX Mboryingabo P. La cysticercose humaine au Rwanda [Humain cysticercosis in Rwanda]. Ann Soc Belg Med Trop. (1972) 52:153–5. 5040226

[53] Tsang V Wilson M. *Taenia solium* cysticercosis: an Unv¡der-recognized but serious public health problem. Parasitol Today. (1995) 11:124–6. 10.1016/0169-4758(95)80175-8

[54] Rottbeck R Nshimiyimana JF Tugirimana P Düll UE Sattler J Hategekimana JC. High prevalence of cysticercosis in people with epilepsy in Southern Rwanda. PLoS Negl Trop Dis. (2013) 7:2558. 10.1371/journal.pntd.0002558 24244783 PMC3828157

[55] National Institute of Statistics of Rwanda. The Third Integrated Household Living Conditions Survey (EICV 3)-Main indicators Report. (2012). Available online at: https://www.statistics.gov.rw/publication/eicv-3-main-indicators-report

[56] Goverment of Rwanda. Environmental and Management plan (ESMP). (2019). Available online at: https://gakenke.gov.rw/fileadmin/templates/document/ESMP__GAKENKE.pdf

[57] Ash LR Oriel TC Savioli L. Bench aids for the diagnosis of intestinal parasites. World Health Org. (1996) 54:548. 10.4269/ajtmh.1996.54.5.TM0540050548a

[58] Knight WB Hiatt RA Cline BL Ritchie LS. A modification of the formol ether concentration technique for increased sensitivity in detecting *Schistosoma mansoni* eggs. Am J Trop Med Hyg. (1976) 25:818–23. 10.4269/ajtmh.1976.25.818 1008127

[59] Allan JC Craig PS Avila G Flisser A Noval JG. Immunodiagnosis of taeniasis by coproantigen detection. Parasitology. (1990) 101:473–7. 10.1017/S0031182000060686 2092303

[60] Gonzalez AE García HH Gilman RH Tsang VCW. Control of *Taenia solium*. Acta Tropica. (2003) 87:103–9. 10.1016/S0001-706X(03)00025-1 12781384

[61] Bustos JA Rodriguez S Jimenez JA Moyano LM Castillo Y Ayvar V. Detection of *Taenia solium* taeniasis coproantigen is an early indicator of treatment failure for taeniasis. Clin Vaccine Immunol. (2012) 19:570–3. 10.1128/CVI.05428-11 22336287 PMC3318286

[62] Tsang VCW Brand JA Boyer AE. An enzyme-linked immunoelectrotransfer blot assay and glycoprotein antigens for diagnosing human cysticercosis (*Taenia solium*). J Infect Dis. (1989) 159:50–9. 10.1093/infdis/159.1.50 2909643

[63] Gonzalez AE Cama V Gilman RH Tsang VCW Pilcher JB Chavera A. Prevalence and comparison of serologic assays, necropsy, and tongue examination for the diagnosis of porcine cysticercosis in Peru. Am J Trop Med Hyg. (1990) 43:194–9. 10.4269/ajtmh.1990.43.194 2389823

[64] Tsang VCW Pilcher JA Zhou W Boyer AE Kamango-Sollo EIP Rhoads ML. Efficacy of the immunoblot assay for cysticercosis in pigs and modulated expression of distinct IgM/ IgG activities to *Taenia solium* antigens in experimental infections. Vet Immunol Immunopathol. (1991) 29:69–78. 10.1016/0165-2427(91)90053-F 1949584

[65] Garcia HH Harrison LJS Parkhouse RME Montenegro T Martinez SM Tsang VCW. A specific antigen-detection ELISA for the diagnosis of human neurocysticercosis. Trans R Soc Trop Med Hyg. (1998) 92:411–4. 10.1016/S0035-9203(98)91069-0 9850394 PMC3025427

[66] Garcia HH. Serological diagnosis and follow-up of severe neurocysticercosis using HP10 antigen detection: commentary. Nat Clin Pract Neurol. (2007) 3:488–9. 10.1038/ncpneuro0563 17637728

[67] Brandt JRA Geerts S Deken R De Kumar V Ceulemans F Brijs L. A monoclonal antibody-based ELISA for the detection of circulating excretory-secretory antigens in *Taenia saginata* cysticercosis. Int J Parasitol. (1992) 22:471–7. 10.1016/0020-7519(92)90148-E 1644522

[68] Van Kerckhoven I Vansteenkiste W Claes M Geerts S Brandt J. Improved detection of circulating antigen in cattle infected with *Taenia saginata* metacestodes. Vet Parasitol. (1998) 76:269–74. 10.1016/S0304-4017(97)00226-4 9650864

[69] Dorny P Phiri IK Vercruysse J Gabriel S Willingham AL Brandt J. A Bayesian approach for estimating values for prevalence and diagnostic test characteristics of porcine cysticercosis. Int J Parasitol. (2004) 34:569–76. 10.1016/j.ijpara.2003.11.014 15064121

[70] Zea-Vera A Cordova EG Rodriguez S Gonzales I Pretell EJ Castillo Y. Parasite Antigen in Serum Predicts the Presence of Viable Brain Parasites in Patients With Apparently Calcified Cysticercosis Only. (2013). Available online at: https://academic.oup.com/cid/article-abstract/57/7/e154/337081 (accessed December 14, 2020). 10.1093/cid/cit422 PMC3765011 23788241

[71] Mupfasoni D Karibushi B Koukounari A Ruberanziza E Kaberuka T Kramer MH. Polyparasite helminth infections and their association to anaemia and undernutrition in Northern Rwanda. PLoS Negl Trop Dis. (2009) 3:517. 10.1371/journal.pntd.0000517 19753110 PMC2737105

[72] Emile N Bosco NJ Karine B. Prevalence of intestinal parasitic infections and associated risk factors among Kigali Institute of Education students in Kigali, Rwanda. Trop Biomed. (2013) 30:718–26. Available online at: http://www.msptm.org/files/718_-_726_Bernard_Karine.pdf 24522143

[73] Rujeni N Morona D Ruberanziza E Mazigo HD. Schistosomiasis and soil-transmitted helminthiasis in Rwanda: an update on their epidemiology and control. Infect Dis Poverty. (2017) 6:8. 10.1186/s40249-016-0212-z 28245883 PMC5331630

[74] Parkhouse RME Rojas R G Aguilar CM Medina C Ferrer E Cortez Alcovedes MM. Diagnosis of taeniosis in rural venezuelan communities: preliminary characterization of a *Taenia solium* specific monoclonal (VP-1) coproantigen ELISA. Acta Trop. (2020) 207:105445. 10.1016/j.actatropica.2020.105445 32224076

[75] García HH González AE Evans CA Gilman RH. Taenia solium Cysticercosis. (2003). Available online at: www.thelancet.com 10.1016/S0140-6736(03)14117-7 PMC3103219 12932389

[76] Jayashi CM Arroyo G Lightowlers MW García HH Rodríguez S Gonzalez AE. Seroprevalence and risk factors for *Taenia solium* cysticercosis in rural pigs of Northern Peru. PLoS Negl Trop Dis. (2012) 6:1733. 10.1371/journal.pntd.0001733 22816002 PMC3398967

[77] Schantz PM Moore AC Muñoz JL Hartman BJ Schaefer JA Aron AM. Neurocysticercosis in an orthodox Jewish community in New York City. N Engl J Med. (1992) 327:692–5. 10.1056/NEJM199209033271004 1495521

[78] Assana E Lightowlers MW Zoli AP Geerts S. *Taenia solium* taeniosis/cysticercosis in Africa: risk factors, epidemiology and prospects for control using vaccination. Vet Parasitol. (2013) 195:14–23. 10.1016/j.vetpar.2012.12.022 23312868

[79] Gweba M Faleke OO Junaidu AU Fabiyi JP Fajinmi AO. Some risk factors for *Taenia solium* cysticercosis in semi-intensively raised pigs in Zuru, Nigeria. Vet Ital. (2010) 46:57–67. Available online at: https://www.izs.it/vet_italiana/2010/46_1/57.htm 20391368

