# Addendum: Evidence for transmission of *Taenia solium* taeniasis/cysticercosis in a rural area of Northern Rwanda

**DOI:** 10.3389/fvets.2025.1579712

**Published:** 2025-04-08

**Authors:** Lucrecia Acosta Soto, Lucy Anne Parker, María José Irisarri-Gutiérrez, Javier Arturo Bustos, Yesenia Castillo, Erika Perez, Carla Muñoz-Antoli, José Guillermo Esteban, Héctor Hugo García, Fernando Jorge Bornay-Llinares

**Affiliations:** ^1^Área de Parasitología del Departamento de Agroquímica y Medioambiente, Universidad Miguel Hernández de Elche, Alicante, Spain; ^2^Departamento de Salud Pública Historia de la Ciencia y Ginecología, Universidad Miguel Hernández de Elche, Alicante, Spain; ^3^Centro de Investigación Biomédica en Red de Epidemiología y Salud Pública (CIBERESP), Madrid, Spain; ^4^Área de Parasitología, Dpto. Farmacia y Tecnología Farmacéutica y Parasitología, Facultat de Farmàcia, Universitat de València, Valencia, Spain; ^5^Dpto. de Ciencias de la Salud, Facultad de Ciencias Biomédicas, Universidad Europea de Madrid, Madrid, Spain; ^6^Cysticercosis Unit, Instituto Nacional de Ciencias Neurológicas, Lima, Peru; ^7^Center for Global Health, Universidad Peruana Cayetano Heredia, Lima, Peru

**Keywords:** *Taenia solium*, taeniasis, cystcercosis, children, Gakenke, Rwanda

Our research group published a brief report in the Journal in April 2021 (doi: 10.3389/fvets.2021.645076) describing the *Taenia solium* taeniosis and cysticercosis prevalence in a Rural area of Rwanda (Gakenke). This study provided evidence of the highest cysticercosis prevalence reported in Rwanda in children to date.

In the months following publication, a comment drew the attention of the editorial board to a number of issues, including the ethical implications of the study [no local institutional review board (IRB) approval was obtained], the lack of Rwandan researchers associated with the study, and the means by which the authors were able to access the communities and schools for data collection (1). In light of the aforementioned considerations, the authors wish to provide some clarifications in this regard.

The ethical approval for the study was granted from an international rather than local IRB. The Miguel Hernández University has been collaborating since the late 1990s in Rwanda, mainly in the Gakenke district, Northern Province. Indeed, the close relationship between Spain and Rwanda has resulted in the establishment of a Miguel Hernández University Chair in Rwanda since 2016 whose objective is to facilitate development cooperation actions in the African country, with a particular focus on the areas of health, research and education. These actions are carried out through the construction or reform of infrastructure, as well as through the provision of teaching and health support (2).

The procedures published in the brief report were carried out at the request of the local Rwandan authorities within the scope of this development cooperation initiative. Regarding why approval from a local ethics committee was not obtained, we were unaware of the establishment of the National Ethics Committee of Rwanda just 3 years earlier. Instead, ethical approval was obtained from the Experimental Research Commission on Ethics from University Miguel Hernández de Elche (Spain) (Ref: DF-MPA001-11), authorization was obtained from the school management and from the ecclesiastical authority, responsible for the school, as well as from the mayor of the city. All of the people responsible for the schoolchildren were duly informed in person at the school, and participation in the study was voluntary and only after the parents or guardians signed an informed consent. H. Nsabo was the local translator who helped us with this aspect, providing the informed consent in Kinyarwanda and she is named in the acknowledgments section as “Jacinte” (3). The hematological and coproparasitological study was carried out together with the technical staff of the Nemba Hospital laboratory (A. Babukiyehe, JD. Mbonigaba and A. Nibishara), with the authorization of the hospital director. Treatment was provided to all children who tested positive for intestinal parasites, free of charge, and in collaboration with Nemba Hospital. Although there were no Rwandan researchers who met the criteria for authorship of this current publication, the collaboration of the technical staff is acknowledged in the acknowledgments section.

With this in mind, we must acknowledge that the procedures that gave rise to this international publication were first and foremost carried out as part of a University Development Cooperation intervention, as mentioned above (with IRB approval and authorization from local authorities), rather than a formal research project aiming to produce publications in scientific journals. This is the main explanation as to why no there was no attempt on our part to inquire about the existence of a national IRB for health research in the country, which ultimately led to a formal research protocol not being submitted to the national IRB.

The surprising finding of *Taenia* spp. eggs in the feces of two schoolchildren, made it necessary to identify the species involved in order to confirm/rule out the possible occurrence of cysticercosis in the region (4). One of the principal findings of our study was the identification of active cases of neurocysticercosis in 38% of seropositive schoolchildren. Neurocysticercosis (NCC) represents the most prevalent parasitic disease affecting the human nervous system and is the most common aetiological factor for epilepsy in low-income countries (5). This disease at the time of the study had not been detected in Rwanda, although it had been detected in all neighboring countries (6). Certainly, the delay between collection of samples, July and September 2011, and the publication, April 2021, is large; thus, new studies are necessary to evaluate an updated prevalence of cysticercosis. Nonetheless, the findings published in the brief report were shared immediately at local level as part of the University Development Cooperation programme, and it was later deemed important to disseminate the findings to the wider scientific community, which explains in part, the delay between performing the procedures and wider dissemination of the findings as an international publication.

In conclusion, this publication shared an important finding for Rwandan public health, since cysticercosis must be included in the differential diagnosis of neurological diseases, especially epilepsy. While we acknowledge that the initiative that led to this publication could have been strengthened by integrating it as a formal research project from the outset with comprehensive local IRB oversight and an equitable partnership with Rwandan co-researchers, we emphasize that the study followed international ethical standards, including informed consent and measures to protect confidentiality during data processing, as would be expected for any international health intervention. With regards to the international research publication, there was a significant delay between the procedures described and publication. We had to carefully balance the ethical considerations of publishing the findings in these conditions vs. withholding these critical findings from an international audience. We believe that sharing these findings at a wider level contributes valuable insights to public health in Rwanda and beyond, while reaffirming our commitment to improving our approach in future studies.
