# Expression of Concern: Evidence for transmission of *Taenia solium* taeniasis/cysticercosis in a rural area of Northern Rwanda

**DOI:** 10.3389/fvets.2025.1593546

**Published:** 2025-04-08

**Authors:** Frontiers Editorial Office

**Affiliations:** Frontiers Media SA, Lausanne, Switzerland

**Keywords:** *Taenia solium*, taeniasis, cystcercosis, children, Gakenke, Rwanda

After publication, concerns involving ethics approval were raised. An investigation according to COPE guidelines was launched, and clarification was requested from the Ethics Committee at the University Miguel Hernandez at Elche (Spain). After an internal investigation, the Ethics Committee at the University Miguel Hernandez confirmed that favorable ethics approval was issued by said Committee, as the author's declaration of responsibility was deemed sufficient at the time. The Ethics Committee also expressed difficulties reaching a firm conclusion due to the unavailability of the required research project documentation.

An addendum has now been published where the authors and the institution further clarify ethical considerations involving this project: Acosta Soto L, Parker LA, Irisarri-Gutiérrez MJ, Bustos JA, Castillo Y, Perez E, Muñoz-Antoli C, Esteban JG, García HH and Bornay-Llinares FJ (2025) Addendum: Evidence for transmission of *Taenia solium* taeniasis/cysticercosis in a rural area of Northern Rwanda. *Front. Vet. Sci*. 12:1579712. doi: 10.3389/fvets.2025.1579712.

